# Subclavian artery-esophageal fistula after placement of a self-expanding metal stent in a patient with esophagogastric anastomosis stenosis

**Published:** 2016

**Authors:** Mohammadreza Seyedmajidi, Maryam Tavassoli, Mojtaba Kianti, Jamshid Vafaeimanesh

**Affiliations:** 1 Golestan Research Center of Gastroenterology and Hepatology-GRCGH, Golestan University of Medical Sciences, Gorgan, Iran.; 2 Clinical Research Development Center, Department of Internal Medicine, Qom University of Medical Sciences, Qom, Iran.

**Keywords:** Subclavian artery-esophageal fistula, Metal stent; Esophagogastric anastomosis, Massive hematemesis.

## Abstract

**Background:**

There have been reports on stent-related vascular erosions about patients with benign or malignant stenosis of the esophagus who received endoscopic stent insertion for palliative intention for oral intake.

**Case presentation:**

A 61-year-old woman with esophageal cancer located in the middle part of esophagus was treated with esophagectomy. Two years following the surgery, malignant stenosis recurred in the esophagogastric anastomosis. A non-covered self-expanding metal stent (10 cm length with a diameter of 18 mm at expanded state) was inserted. Three months later, a massive hematemesis with subsequent hemorrhagic shock developed from the proximal end of the stent which resulted in the final diagnosis of arterioesophageal fistula on the left subclavian artery. An endovascular repair using a stent graft for the left subclavian artery via the right common iliac artery was performed and the patient remained well until discharge.

**Conclusion:**

Increase in the treatment of esophageal strictures by stent insertion increases the risk of stent-related vascular fistula. These complications should be considered in any patients with massive upper gastrointestinal bleeding.

Standard therapy for early esophageal squamous cell carcinoma is surgical resection or neo-adjuvant chemoradiotherapy followed by resection. In advanced stages, palliative therapy such as endoscopic placement of a stent is a safe and effective procedure to relieve dysphagia (1). Esophagoarterial fistula is a very rare life-threatening complication after esophagectomy (2). The first case of a fistula between the esophagogastric anastomosis and the thoracic aorta was reported in 1946. The patient died from massive bleeding and the results of autopsy confirmed the diagnosis of an aortoesophageal fistula with involvement of the anastomosis (3). In contrast to esophagoarterial fistula, anastomotic leakage is a frequent complication after esophagectomy. Due to advances in surgical technique and perioperative management, the outcomes of surgery have considerably improved with in-hospital mortality decreasing from 29 to 7.5% (4–6). However, postoperative esophagoarterial fistula after esophagectomy has remained an infrequent but fatal complication (7). On the other hand, there have been reports on stent-related vascular erosion. Several reports have shown development of complications in patients with benign or malignant stenosis of the esophagus who received endoscopic stent insertion for palliative intention for oral intake (8-13). 

We describe a complication of a self-expanding metal stent in a patient with recurrent malignant stenosis after esophagectomy due to esophageal carcinoma.

## Case Presentation

A 61-year-old woman with an esophageal squamous-cell carcinoma at stage T2N0, in the middle part of the esophagus with no lymph node involvement was treated with an esophagectomy and gastric pull-up. Two years following the surgery, dysphagia developed and malignant stenosis recurred in the esophagogastric anastomosis. Because of patient disinclination, chemoradiotherapy was not done and a non-covered self-expanding metal stent (10 cm long with a diameter of 18 mm when expanded) was inserted. Three months later, severe hematemesis and hemodynamic shock developed. 

**Figure 1:**
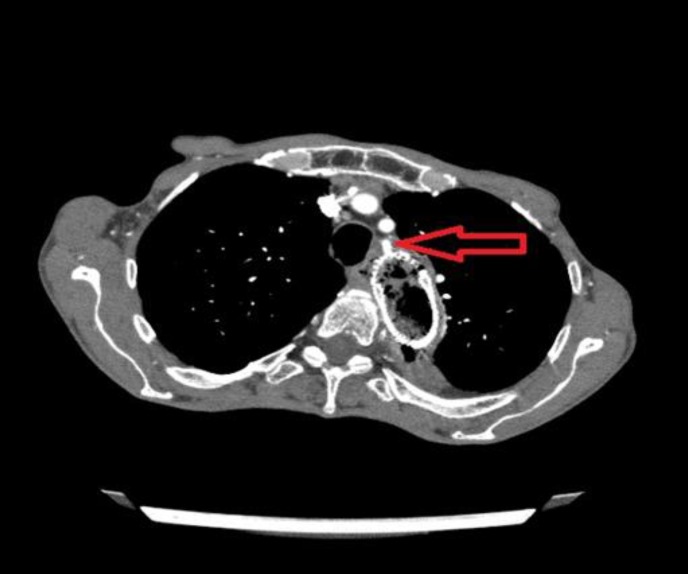
Computed tomography demonstrating an arterioesophageal fistula (red arrow) on the left subclavian artery where the esophageal stent touched

**Figure 2:**
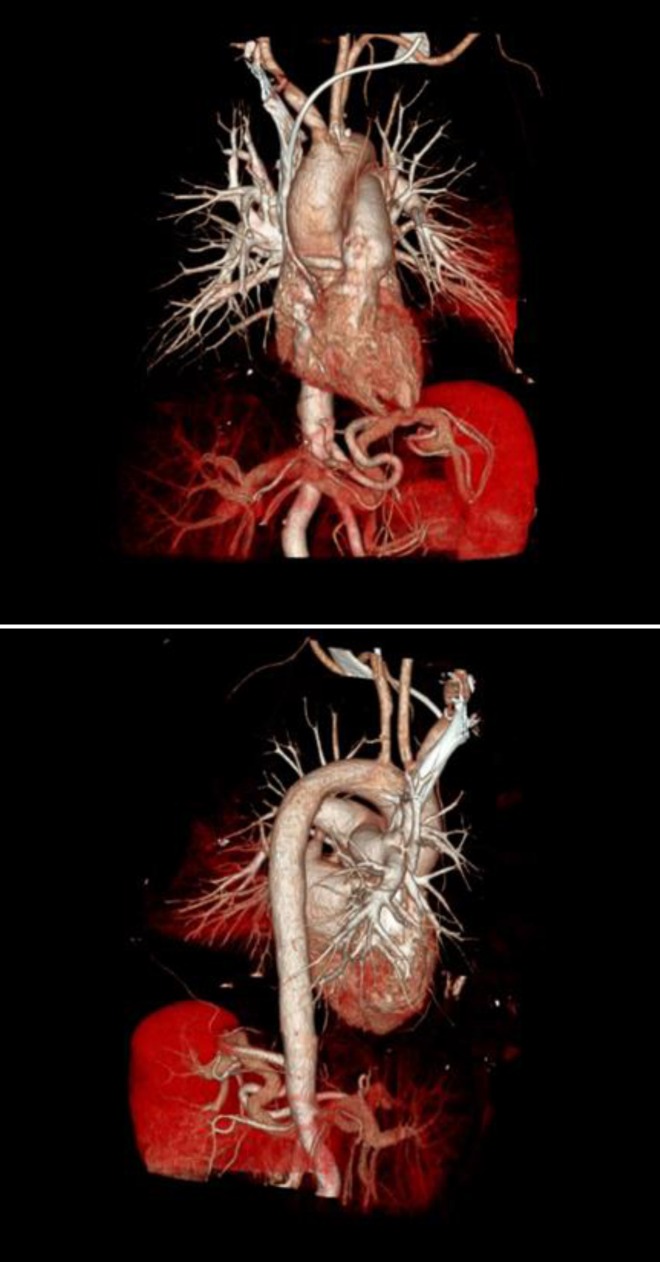
CT angiography of arterioesophageal fistula on the left subclavian artery

Endoscopic examination revealed massive hemorrhage from the proximal end of the stent. An endoscopic intervention to stop the bleeding was unsuccessful. Computed tomography (CT) and CT-angiography demonstrated an arterioesophageal fistula on the left subclavian artery in contact with the esophageal stent (figures 1 and 2). The cardiovascular surgeons performed emergency, endovascular repair and a stent graft was used for the left subclavian artery via the right common iliac artery. The patient remained well without bleeding and could eat one week after arterial repair.

## Discussion

Esophagoarterial fistula is an infrequent complication after esophagectomy (1). The first successful surgical intervention in a case of postoperative aortoesophageal fistula was described by Maillard et al. in 1967 (14). They reported four patients with aortoesophageal fistula after thoracoabdominal partial esophagectomy. Apparently fistula had simultaneously generally improved outcome after esophageal surgery became a very rare complication that had only sporadically been seen. Nearly all reported fistulas originated from an insufficient anastomosis (1). The report of stent-related vascular erosions has been mostly reported in patients with benign or malignant strictures of the esophagus who received stent insertion in palliative intention for oral intake. Erosion of the stent through the esophageal wall into the aortic arch was reported after prolonged stent placement of several months as well as after short placement of only a few days (9-12). Also, stent-related erosions of other major blood vessels were reported (8). Aortoesophageal fistulas can develop after esophagogastrectomy in patients with normal aortic anatomy. In a review of 500 cases of aortoesophageal fistula, 15 were presumed to be due to esophagogastrectomy. All 15 of these cases occurred between 12 and 40 days postoperatively. Among the 500 reported cases of aortoesophageal fistula, three cases were believed to be due to atherosclerosis and ulceration of the atheroma (15, 16). Nearly all of patients had received no neoadjuvant therapy. Therefore neoadjuvant chemotherapy or chemoradiotherapy does not provide an explanation for the origin of aortoesophageal fistula, but additional mechanical irritation by the stent may have a contribution in the development of fistula. In conclusion, increase in the treatment of esophageal strictures by means of endoscopic stent insertion puts the risk of stent-related vascular fistula. This fistula is a very rare, but potentially fatal cause of bleeding. Bleeding is often massive and develops. Awareness to life-threatening and often fatal consequences of these conditions are of particular importance. Notwithstanding, the risk of esophagoarterial fistula, stent implantation remains an effective therapy. Further modifications of the design of stents are required to improve their safety.
